# Ecotin-Like ISP of *L. major* Promastigotes Fine-Tunes Macrophage Phagocytosis by Limiting the Pericellular Release of Bradykinin from Surface-Bound Kininogens: A Survival Strategy Based on the Silencing of Proinflammatory G-Protein Coupled Kinin B_2_ and B_1_ Receptors

**DOI:** 10.1155/2014/143450

**Published:** 2014-09-10

**Authors:** Erik Svensjö, Larissa Nogueira de Almeida, Lucas Vellasco, Luiz Juliano, Julio Scharfstein

**Affiliations:** ^1^Instituto de Biofísica Carlos Chagas Filho, Universidade Federal do Rio de Janeiro, 21990-400 Rio de Janeiro, RJ, Brazil; ^2^Escola Paulista de Medicina, Universidade Federal de São Paulo, 04044-020 São Paulo, SP, Brazil

## Abstract

Inhibitors of serine peptidases (ISPs) expressed by *Leishmania major* enhance intracellular parasitism in macrophages by targeting neutrophil elastase (NE), a serine protease that couples phagocytosis to the prooxidative TLR_4_/PKR pathway. Here we investigated the functional interplay between ISP-expressing *L. major* and the kallikrein-kinin system (KKS). Enzymatic assays showed that NE inhibitor or recombinant ISP-2 inhibited KKS activation in human plasma activated by dextran sulfate. Intravital microscopy in the hamster cheek pouch showed that topically applied *L. major* promastigotes (WT and Δ*isp*2/3 mutants) potently induced plasma leakage through the activation of bradykinin B_2_ receptors (B_2_R). Next, using mAbs against kininogen domains, we showed that these BK-precursor proteins are sequestered by *L. major* promastigotes, being expressed at higher % in the Δ*isp*2/3 mutant population. Strikingly, analysis of the role of kinin pathway in the phagocytic uptake of *L. major* revealed that antagonists of B_2_R or B_1_R reversed the upregulated uptake of Δ*isp*2/3 mutants without inhibiting macrophage internalization of WT *L. major*. Collectively, our results suggest that *L. major* ISP-2 fine-tunes macrophage phagocytosis by inhibiting the pericellular release of proinflammatory kinins from surface bound kininogens. Ongoing studies should clarify whether *L. major* ISP-2 subverts TLR_4_/PKR-dependent prooxidative responses of macrophages by preventing activation of G-protein coupled B_2_R/B_1_R.

## 1. Introduction

Integrated by 3 serine proteases, factor XII (FXII), factor XI (FXI), and plasma prekallikrein (PK) and by one nonenzymatic cofactor, high molecular weight kininogen (HK), the kallikrein-kinin system (KKS), also referred to as the plasma contact pathway of coagulation, is assembled and activated when the blood comes in contact with negatively charged polymers of endogenous origin or microbial surfaces [1, 2]. Upon binding to these negatively charged structures, the zymogen FXII undergoes a conformational change that endows the unstable proenzyme with limited enzymatic activity. Activated FXII (FXIIa) then cleaves prekallikrein (complexed to the cofactor HK), generating PKa. Reciprocal cleavage reactions between FXIIa and PKa amplify the proteolytic cascade, leading to downstream (i) generation of fibrin via the FXIIa/FXIa-dependent procoagulative pathway, (ii) release of the internal bradykinin (BK) moiety of HK by PKa. Once liberated, the short-lived BK induces vasodilation and increases microvascular permeability through the activation of bradykinin B_2_ receptors (B_2_R) expressed in the endothelium lining [1]. In addition, the multifunctional PKa generates plasmin, an effector of fibrinolysis, and cleaves native C3 of the complement system C3 [3, 4].

Although HK is classically regarded as the parental precursor of proinflammatory kinins, the cleaved form of HK (HKa), a disulfide linked two-chain structure, has additional biological functions. For example, it has been reported that HKa reduces neutrophil adhesive functions upon binding to *β*2-integrin Mac-1 (CR3, CD11b/CD18, *α*M*β*2) [1, 5]. More recently, Yang et al. [6] appointed HK/HKa as the plasma-borne opsonins that drive efferocytosis of apoptotic cells via plasminogen activator receptor (uPAR)/RAC1-pathway. After binding to phosphatidyl serine (PS) exposed by apoptotic neutrophils, the surface-bound HK binds to uPAR before switching off proinflammatory responses of macrophages [6]. In contrast to this novel immunoregulatory function, HK (and low molecular weight kininogen, LK) are traditionally viewed as precursors of proinflammatory kinins. Once leaked into extravascular tissues, the plasma-borne HK/LK undergo proteolytic cleavage by tissue kallikrein, releasing the B_2_R agonist lysyl-BK (LBK) in inflammatory exudates [1]. It is noteworthy that oxidized forms of kininogens may release bioactive kinins as result of cooperation between neutrophil elastase (NE) and mast cell tryptase [7, 8]. Acting as paracrine hormones, the short-lived kinins (BK or LBK) swiftly activate G-protein coupled bradykinin B_2_ receptors (B_2_R), a subtype of receptor constitutively expressed by endothelial cells, nociceptive neurons, macrophages, and DCs [1, 9–11]. The long-range signaling activity of intact kinins is controlled by kinin-degrading metallopeptidases, such as the angiotensin converting enzyme (ACE/kininase II) [1]. In addition, the liberated kinin peptides are metabolized by kininase I (carboxypeptidase N or M); removal of the C-terminal Arg residue generates des-Arg-kinins, the high-affinity ligands of B_1_R, a GPCR subtype whose expression is strongly upregulated in injured/inflamed tissues [12].

During the last decade, research conducted in our laboratory showed that kinins proteolytically released in peripheral sites of* T. cruzi* or* Leishmania chagasi *infection reversibly couple inflammation to antiparasite immunity [13–17]. Another interesting twist came from studies showing that activation of the contact system/KKS promotes bacterial entrapment within fibrin meshes, thus providing a physical barrier against the systemic spread of microbial pathogens [18]. To this date, however, it is unclear whether the contact pathway modulates immunity at early stages of cutaneous leishmaniasis. Intravital microscopy studies conducted in the mouse ear model of* L. major* infection [19–21] have shown that infiltrating neutrophils engulf the promastigotes before expressing the apoptotic markers required for efferocytosis by dermal DCs. After internalizing the parasitized/apoptotic neutrophils, the dermal DCs are no longer capable of steering protective TH1-responses in the draining lymph node [19–21]. Although efferocytosis has strong impact on DC function and TH development in* L. major* infection, independent studies showed that macrophage clearance of apoptotic neutrophils may either induce pro- or anti-inflammatory responses in NE-dependent manner, the intracellular fate of the parasite being influenced by the host genetic background [22, 23].

In natural infection by blood-feeding arthropods, insect proboscis inevitably causes bleeding, which then causes the mixing of plasma and sandfly saliva substances with parasites deposited in the injured dermis [24]. Interestingly,* Phlebotomy duboscq*, a vector of* Leishmania* species, contains high levels of a salivary protein (PdSP15) that inhibits the contact pathway [25] by binding to negatively charged polymers of endogenous origin—such as platelet-derived polyphosphates [2, 18, 26]. Considering that activation of the procoagulative contact system induces microvascular leakage through PKa-mediated release of BK, it is conceivable that sandfly-transmitted* Leishmania* promastigotes have evolved the means to subvert the innate effector function of the kinin pathway at early stages of infection.

The current study was motivated by the recent discovery that* Leishmania* has three genes encoding ecotin-like inhibitors of serine peptidases (ISPs) [27]. Previous studies with the archetype of the family* Escherichia coli* ecotin [28] showed that this inhibitor targets neutrophil elastase (NE) [29]—a member of the trypsin-fold serine peptidases of clan PA/family S_1A_. After noting that the* Leishmania* genome [30] lacks these endogenous serine peptidase targets, Eschenlauer et al. [27] predicted that* L. major* ISPs might target S_1A_-family serine peptidases expressed by cells of the innate immune system, such as NE, tryptase, and cathepsin G [30]. In a series of elegant studies, Eschenlauer et al. [27] and Faria et al. [31, 32] addressed this issue using* L. major* lines lacking ISP2 and ISP3 (Δ*isp*2/3). After studying the outcome of interactions between* L. major* promastigotes and elicited macrophages, these authors found that these phagocytes internalized the Δ*isp*2/3 promastigotes far more efficiently than ISP-expressing wild-type (WT) parasites [27, 31] and linked the upregulated CR3-dependent phagocytosis of the Δ*isp*2/3* L. major* mutants to NE-dependent activation of innate immunity via the TLR_4_/PKR/TNF-*α*/IFN-*β*, a prooxidative pathway that limits intracellular parasite survival [31, 32]. Notably, the phenotype of Δ*isp*2/3 promastigotes was reversed by supplementing the macrophage cultures with purified (recombinant) ISP-2 or with the synthetic NE inhibitor (MeOSuc-AAPV-CMK), at the onset of infection [31]. Based on these collective findings, these authors suggested that ISP-2 expressing* L. major* promastigotes might downmodulate phagocytosis and limit microbicidal responses of macrophages by preventing NE-dependent activation of TLR_4_ [31, 32]. More recently, we have documented that macrophages internalize and limit intracellular* T. cruzi* growth in resident macrophages through activation pathways forged by the cross-talk between bradykinin B_2_ receptors and C5a receptors [33]. Intrigued by the similarities that exist between the phenotype of the* L. major *Δ*isp*2/3 mutant and* L. chagasi* promastigotes [15] and* T. cruzi* trypomastigotes (Dm28 strain) [33, 34], in the current work we interrogated whether ISP-expressing* L. major* and the ISP-2 Δ*isp*2/3 mutants differ in their ability to activate the KKS* in vivo* and* in vitro*. Using intravital microscopy, we first showed that* L. major* promastigotes topically applied to the hamster cheek pouch potently activate the KKS extravascularly, irrespective of presence/absence of ISP. In the second part of this study, we present evidence indicating that ISP-expressing* L. major* may subvert innate immunity by targeting kinin-releasing serine proteases (S_1A_ family) exposed at the cell-surface of macrophages.

## 2. Materials and Methods

### 2.1. Parasites

*L. major* Promastigotes of Friedlin (MHOM/JL/80/Friedlin) were grown in modified Eagle's medium (HOMEM, Sigma) supplemented with 10% heat-inactivated fetal bovine serum (FBS, Gibco) at 25°C, as previously described [31, 32]. Suspensions of promastigotes were washed twice with PBS before being used either* in vitro* or* in vivo*.* Leishmania major* deficient in ISP2 and ISP3 (Δ*isp*2/3) were generated as previously described by Eschenlauer et al. [27]. The following antibiotics were used at the indicated concentration for the selection of transfectants: 50 mg/mL hygromycin B (Roche), 25 mg/mL G418 (Invitrogen), 10 mg/mL phleomycin (InvivoGen), and 50 mg/mL puromycin dihydrochloride (Calbiochem).

### 2.2. Intravital Digital Microscopy

Syrian hamsters, 3-month-old males, were maintained and anesthetized according to regulations given by the local ethical committee (IBCCF, protocol-014, 23/02/2008). Altogether 65 hamsters (114 ± 18 g) (Anilab, São Paulo, Brazil) were used. Anesthesia was induced by intraperitoneal injection of sodium pentobarbital 3% that was supplemented with i.v. *α*-chloralose (2.5% W/V, solution in saline) through a femoral vein catheter. A tracheal cannula (PE 190) was inserted to facilitate spontaneous breathing and the body temperature was maintained at 37°C by a heating pad monitored with a rectal thermistor. The hamster cheek pouch (HCP) was prepared and used for intravital microscopy as previously reported [34, 35]. The microcirculation of the HCP was observed using an Axioskop 40 microscope, objective 4x, and oculars 10x equipped with a LED light source Colibri (Carl Zeiss, Germany) and appropriate filters (490/520 nm and 540/580 nm, rhodamine) for observations of fluorescence in epiluminescence. A digital camera, AxioCam HRc, and a computer with the AxioVision 4.4 software program (Carl Zeiss, Germany) were used for image analysis of arteriolar diameter and total fluorescence in a representative rectangular area (5 mm^2^) of the prepared HCP. Fluorescence was recorded for 30 min prior to experimental interventions to secure normal blood flow and unaltered vascular permeability and the fluorescence measured at 30 min after FITC-dextran (FITC-dextran 150 kDa, 100 mg/kg bodyweight, TdB Consultancy, Uppsala, Sweden) injection was adjusted to 2000 fluorescent units (RFU = Relative Fluorescent Units) for statistical reasons. Leukocytes were labeled* in vivo* by injecting rhodamine 100 *μ*g/kg b.w i.v. (10 min prior to experimental interventions), reinforced by injection of the same tracer at 10 *μ*g/kg b.w. every 10 min until 60 min. The recorded fluorescence at 10 min after rhodamine injection in each experiment was adjusted to 3000 fluorescent units (RFU) for statistical reasons. Two images of exactly the same area were recorded at every 5 min interval during the entire experiment. One was used to measure plasma leakage and arteriolar diameter (490/520 nm) and the other to measure total fluorescence of rhodamine-labeled leukocytes in circulation, rolling, adherence, and migration (540/580 nm) in the observed area (5 mm^2^) here defined as leukocyte accumulation. Exposure time was limited to 15 s for each captured image in order to avoid phototoxicity. Following 30 min control period after FITC-dextran injection HCPs were topically exposed to WT* L. major* promastigotes or Δ*isp*2/3 (7.5 × 10^6^/500 *μ*L) during interruption of the superfusion for 10 min. Cromoglycate was injected i.p. (40 mg/kg b.w.) at time of pentobarbital anesthesia and dextran sulfate 500 kDa (TdB Consultancy, Uppsala, Sweden) was injected i.v. (2 mg/kg) prior to parasite application. HOE-140 tested at 0.5 *μ*M and the histamine receptor H1 mepyramine (10 *μ*M) were applied locally via a syringe pump into the superfusion during 10 min prior to application of promastigotes.

### 2.3. Isolation of Peritoneal Macrophages and Invasion Assays

C57BL/6 mice received an intraperitoneal injection of 2 mL of 3% thioglycolate and macrophages were harvested from peritoneal lavage 3 days later. Macrophages were plated on 13 mm coverslips in 24-well plate and after 20 h of incubation at 37°C in complete medium (RPMI + 10% FBS, 100 U/mL penicillin, and 100 *μ*g/mL streptomycin) the nonadherent cells were removed by washing the monolayer of cells with PBS. Invasion assays were performed by adding stationary phase promastigotes to the monolayers at a ratio of 5 : 1 (parasite/macrophage) in medium containing 1 mg/mL albumin from bovine serum (BSA, Sigma). The interaction was performed during 3 h in a humidified chamber containing 5% CO_2_ at 37°C. When indicated, the culture medium was supplemented with 100 nM of B_2_R antagonist (HOE-140, Sigma) or 1 *μ*M of B_1_R antagonist des-Arg^9^-[Leu^8^]-BK (DAL^8^-BK; Sigma), 5 minutes before addition of parasites. After interaction, extracellular promastigotes were removed by washing the monolayers twice with PBS, which were then fixed with Bouin overnight and stained with Giemsa (Merck). The number of intracellular amastigotes was determined by counting at least 100 cells per replicate under the light microscope. All assays were done in triplicates and results were expressed as mean values ± SD.

### 2.4. Parasite Surface Staining

Promastigotes were preincubated with PBS-1% BSA for 1 hour to avoid unspecific binding and then incubated for 1 h with monoclonal antibody MBK_3_ (IgG1—1 : 50) or HKH4 (IgG_2a_—1 : 50), kindly provided by Dr. W. Müller-Esterl from Frankfurt University. MBK_3_ recognizes the BK epitope in domain D4 of human and bovine H-/L-kininogens whereas HKH_4_ binds to the D1 domain [36]. Isotype-matched monoclonal antibodies were used as negative controls. Parasites were washed three times with PBS-1% BSA and incubated with secondary fluorescent antibody (FITC—1 : 50) for 30 min at 4°C, protected from light. After washing, the samples were acquired by flow cytometry (FACSCan; BD Biosciences), and data analyses were done with Summit software (Dako Colorado, Inc). Assays were done in duplicates and results are representative of two independent experiments.

### 2.5. Contact Phase Activation of Human Plasma

The activation of FXII/PK in human citrated (platelet free) plasma treated (or not) with dextran sulfate (DXS; 500 kDa, TdB Consultancy) was monitored by spectrofluorimetry as previously described [37], using internally quenched fluorescent substrates whose sequences correspond to the C-terminal (Abz-GFSPFRSVTVQ-EDDnp) or N-terminal flanking region (Abz-MTEMARRPQ-EDDnp m) of BK of mouse kininogen. The hydrolysis of the cleaved substrate Abz-peptidyl-EDDnp (Abz = o-aminobenzoyl and EDDnp = ethylenediamine 2,4-dinitrophenyl) was monitored by measuring the fluorescence at *λ*_ex._ = 320 nm and *λ*_em._ = 420 nm in a Spectramax M5 fluorescence spectrophotometer. The reaction was carried out in PBS, pH 7.4, using citrated human plasma 1 : 20, 4 *μ*M of the Abz-peptidyl-EDDnp substrate and 20 nM of the contact system activator DXS (500 kDa). As internal controls, the plasma was pretreated with the synthetic PKa inhibitor (PKSI-527—5 *μ*M) [38]. Assays with recombinant ISP-2 (kindly supplied by A. P. C. A. Lima) were performed at final concentrations of 142, 177, 240, and 355 nM; the neutrophil elastase (NE) inhibitor MeOSuc-AAPV-CMK (Calbiochem) was tested at 10, 20, and 30 *μ*M. PKSI or recombinant ISP2 and MeOSuc-AAPV-CMK were preincubated with human plasma for 15 min, at 37°C, prior to the addition of DXS and the substrate. Plasma was prepared by centrifugation of blood samples at 2500 g for 20 min at 4°C. After centrifugation, plasma samples were filtered using a 0.2 *μ*m membrane.

### 2.6. Statistical Analyses

Statistical analyses were done using PRISM 5.0 (GraphPad Software). Comparisons of the means of the different groups were done by one-way analysis of variance (ANOVA). When the mean values of the groups showed a significant difference, pairwise comparison was performed with the Tukey test. A *P* value of 0.05 or less was considered to indicate a statistically significant difference. For intravital experiments, we used ANOVA or pairwise* t*-test, when appropriate.

## 3. Results

### 3.1. Analysis of the Dynamics of Inflammation in HCP Topically Sensitized with* L. major* Promastigotes

**Figure 1:**
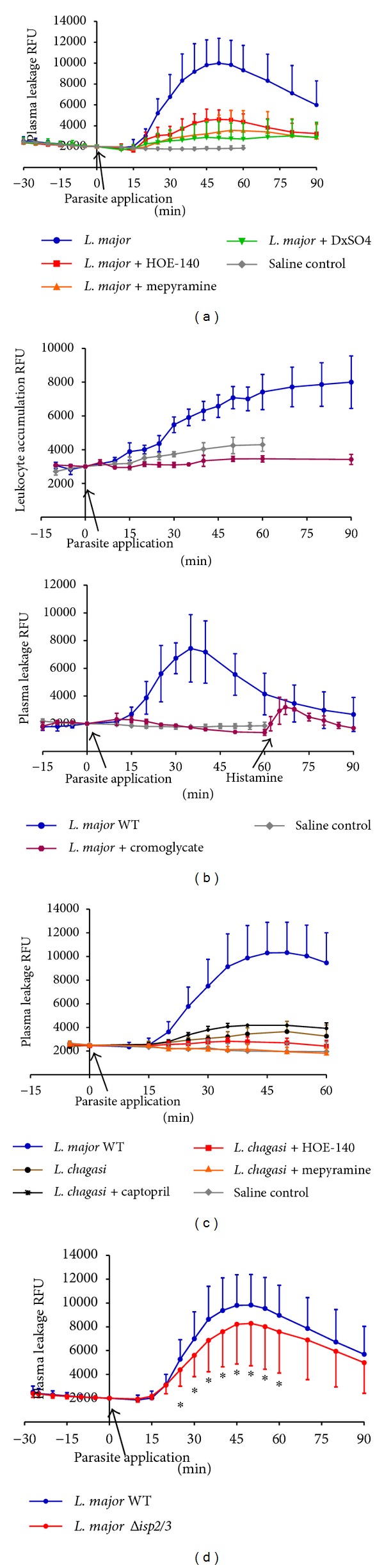
Microvascular plasma leakage and leukocyte accumulation in the HCP sensitized with* Leishmania*. The data represent relative fluorescence units (RFU: mean ± SD) induced by the topical application of* L. major* promastigotes (MHOM/JL/80/Friedlin, 500 *μ*L of 1,5 × 10^7^/mL) on hamster cheek pouch preparations (HCPs) after 30 min of stabilization period without significant increase in RFU. Applications of promastigotes were made during 10 min of interrupted superfusion of the HCPs. (a) Pharmacological interventions. Four groups of HCP were sensitized with* L. major* WT promastigotes, whereas one group (saline control; *n* = 6) served as untreated control. The first group (*n* = 15) corresponds to the positive controls, that is, profile of HCP exposed to* L. major* alone; the second group (*n* = 5) received HOE-140 (0.5 *μ*M) 5 min prior to promastigote application; the third group (*n* = 4) received the antagonist of histamine receptor (H1R) mepyramine (10 *μ*M) 5 min prior to challenge with promastigotes; and the fourth group (*n* = 7) was pretreated (i.v.) with dextran sulfate 500 (DXS-500; 2 mg/kg) at time of FITC-dextran injection. Plasma leakage was significantly reduced (*P* < 0.05) in all experimental groups subjected to pharmacological interventions. (b) Effect of the mast cell stabilizer cromoglycate. Data represent mean values ± SD obtained in HCP sensitized by* L. major* WT (*n* = 4) and a saline control group (*n* = 6). Two hamsters were given cromoglycate 40 mg/kg i.p. at time of anesthesia induction, and this treatment resulted in a complete inhibition of plasma leakage and leukocyte accumulation elicited by* L. major *despite the fact that the HCPs responded to histamine stimuli (4 *μ*M) at the end of the experiment, that is, 60 min after topical application of* L. major* promastigotes. As an internal control, one hamster from the DXS-treated group (*n* = 7, Figure 1(a)) received rhodamine i.v. prior to parasite challenge. Measurements of leukocyte accumulation showed that DXS-500 reduced the* Leishmania* response to levels below the saline control group while plasma leakage decreased to the level of controls depicted in Figure 1(a) (data not shown). (c) Comparative analysis of kinin/B_2_R-driven microvascular plasma leakage induced by different* Leishmania *species. The graph depicts responses evoked by* L. major* WT and* L. chagasi* promastigotes (500 *μ*L de 1,5 × 10^7^/mL).* L. major* WT (blue filled circles, *n* = 19);* L. chagasi* (brown filled circles, *n* = 6);* L. chagasi* + captopril 1 *μ*M (black crosses, *n* = 4);* L. chagasi* + o.5 *μ*M HOE-140 (red squares, *n* = 4);* L. chagasi* + 10 *μ*M mepyramine (orange triangles, *n* = 4); and saline control (grey diamonds, *n* = 3). The maximal microvascular response to* L. major* was 5-fold higher than* L. chagasi* at 50 min after parasite application. The tests involving pharmacological interventions in HCP sensitized with* L. chagasi* groups were different (*P* < 0.05) from the* L. chagasi* control at 40 min. (d) Microvascular plasma leakage elicited by* L. major *Δ*isp*2/3. The data represent mean values ± SD. One group represents the microvascular responses evoked by WT* L. major* (MHOM/JL/80/Friedlin, *n* = 19) whereas the second group represents responses induced by* L. major *Δ*isp*2/3 (*n* = 16). The plasma leakage induced by WT versus Δ*isp*2/3 promastigotes was significantly different (*P < 0.05) between 25 and 60 min after topical application of the pathogens.

**Figure 2:**
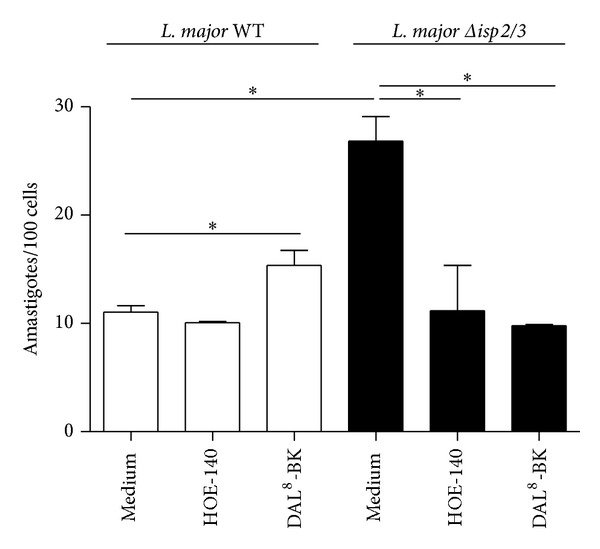
Differential role for bradykinin receptors in the phagocytic response of macrophages infected by WT* L. major* promastigotes and Δ*isp*2/3 mutants. Thioglycolate elicited (peritoneal) macrophages were incubated in medium containing 1 mg/mL BSA in the presence or absence of 100 nM of HOE-140 or 1 *μ*m of des-Arg^9^-[Leu^8^]-BK (DAL^8^-BK). Promastigotes were added (parasite/cell ratio 5 : 1) and incubated for 3 h at 37°C. White bars represent* L. major* WT and black bars represent Δ*isp*2/3. Data represent numbers of intracellular amastigotes per 100 macrophages (means ± SD) for triplicates and represent two different experiments (*P < 0.05).

**Figure 3:**
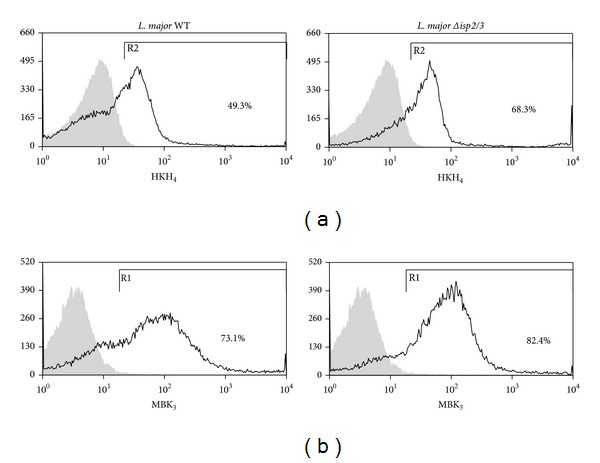
Evidence of differential display of kininogens and BK epitopes on surface of WT* L. major* versus Δ*isp*2/3 promastigotes. WT and Δ*isp*2/3 promastigotes were washed 3x before incubation with mAbs against D1 or D4 (BK epitope) of kininogens, HK/LK (HKH_4_ (a) or MBK_3_ (b), resp.) for 1 h. Unrelated Ab (IgG_2a_ for HKH_4_ or IgG1 for MBK_3_ staining) were used as specificity controls. Binding of primary IgG was assessed by incubating the cells with a secondary FITC-labeled anti-mouse IgG antibody for 1 h. The graphs represent the percentage of HK/LK adsorption and are representative of two independent experiments performed in duplicates.

Intravital microscopy in HCP has provided a wealth of information about the interplay between the KKS and the topically applied pathogens because this method dispenses the use of needles, thus ruling out the influence of bleeding and collateral activation of the contact system in the analysis of microcirculatory parameters. As a starting point in this work, we asked whether the proinflammatory responses evoked by ISP-expressing* L. major* promastigotes or ISP-deficient parasites were comparable. Our results (Figures 1–3) revealed that* L. major* (WT) promastigotes induced a very robust and reversible microvascular leakage that was detectable at 20 min and reached its maximal value 45 min after pathogen application (Figures 1(a)–1(c)). In 4 out of 15 experiments we measured leukocyte accumulation in and around postcapillary venules and noted that these circulating cells were promptly mobilized locally, the response being detectable up to 90 min after pathogen application (Figure 1(b)). The temporal course and dynamics of the plasma leakage response evoked by WT promastigotes were quite different from the classical responses elicited by BK, histamine, or leukotrienes, all of which cause a maximal increase within 10 min and reversed to steady-state conditions within 30 min [39, 40]. Intriguingly, we found that the leakage responses evoked by* L. major* (WT) promastigotes were generally more robust than those induced by the same inoculum of* L. donovani* promastigotes (Figure 1(c)) [16] or* T. cruzi* (tissue culture trypomastigotes, Dm28c strain) [35]. Akin to the findings made in the above-mentioned studies, we found that topically applied HOE-140 (B_2_R antagonist) markedly reduced* L. major* (WT-) induced plasma leakage (Figure 1(a)).

Considering that mast cells are innate sentinel cells strategically localized in the perivascular tissues, we next interrogated whether* L. major* promastigotes might evoke plasma leakage in mast cell-dependent manner. As shown in Figure 1(a), the topical addition of mepyramine (histamine-1-receptor blocker) markedly inhibited the macromolecular leakage induced by* L. major* promastigotes. In a limited series of studies, we found that cromoglycate, a well-known mast cell stabilizer, abolished the* L. major*-induced leakage of plasma (Figure 1(b), lower panel). Of further interest, cromoglycate prevented leukocyte accumulation in the parasite-laden microvascular beds, reducing this parameter to levels below controls (Figure 1(b), top panel). It is noteworthy that the doses of mepyramine and HOE-140 that were topically added to the HCP at the onset of infection were sufficient to block the leakage induced by standard solutions of histamine (4 *μ*M) and BK (0.5 *μ*M) [35]. Further expanding this investigation, we next explored the possibility that the microvascular leakage elicited by* L. major* promastigotes requires the participation of circulating neutrophils. To this end, we injected separate group of hamsters intravenously with DXS, a negatively charged polymer (500 kDa) and found that it profoundly inhibited plasma leakage induced by* L. major* promastigotes. Although DXS was initially thought to inhibit neutrophil-dependent microvascular permeability by blocking endothelial interaction with neutrophil-derived cationic proteins [41–44], there is now awareness that these effects might result from DXS-mediated activation of the KKS, a systemic reaction that leads to hypotension as result of excessive BK formation [45].

Finally, we sought to compare the microvascular responses elicited by WT* L. major* with their counterparts genetically deficient in ISP-2/ISP-3. As shown in Figure 1(d), the dynamics of plasma leakage induced by topically applied Δ*isp*2/3 mutants was similar to that evoked by WT parasites, the peak response being observed at 45–50 min after pathogen application. However, somewhat surprisingly, the Δ*isp*2/3 mutants were 20% less effective in eliciting transendothelial leakage of plasma as compared to WT promastigotes (Figure 1(d)); the difference in their proinflammatory phenotypes (*P* < 0.05) was already noticeable at 25 min after parasite application.

### 3.2. Bradykinin Receptors Selectively Fuel Macrophage Internalization of ISP-Deficient* L. major*

Studies in mice models of acute Chagas disease [14] and visceral leishmaniasis [16] have recently showed that B_2_R-deficient mice exhibited impaired development of type-1 effector T cells, the immune dysfunction of the transgenic strain being ascribed to primary deficiency in the maturation of B_2_R^−/−^ DCs in chagasic mice [14]. In a third study, we examined the role of the kinin pathway in the* in vitro* outcome of macrophage interactions with* L. chagasi* promastigotes [15]. Interestingly, these studies revealed that activation of the kinin/B_2_R pathway may either fuel intracellular parasite outgrowth in splenic macrophages from hamsters, a species that is susceptible to visceral leishmaniasis, or limit parasite survival in thioglycolate-elicited mouse peritoneal macrophages [16]. Motivated by this groundwork, in the next series of experiments we examined the outcome of macrophage interaction (3 h in the absence of serum) with* L. major* promastigotes or Δ*isp*2/3 mutants. Consistent with the phenotypic properties of Δ*isp*2/3 promastigotes originally described by Eschenlauer et al. [27], we found that the phagocytic uptake of these mutants was strongly upregulated as compared to WT promastigotes (Figure 2). Next, we asked whether B_2_R (constitutively expressed) or B_1_R (NF*κ*-B inducible; [35]) contributed to the phagocytic uptake of* L. major*. Infection assays performed in the presence of HOE-140 or DAL^8^-BK (B_1_R antagonist) revealed that none of these GPCR antagonists inhibited macrophage uptake of ISP-expressing (WT)* L. major*. In striking contrast, however, both GPCR antagonists efficiently reduced the phagocytic uptake of Δ*isp*2/3 promastigotes by the phagocytes (resp., to 58% and 63%; Figure 2). For reasons that are not clear, the B_1_R antagonist had a mild but significant stimulatory effect (39% increase compared with medium) on the uptake of WT* L. major.*

### 3.3. Surface Exposure of the BK Epitope Differs in WT and Δ*isp*2/3 Promastigotes

Since the studies of macrophage infection by* L. major* promastigotes were routinely performed in the absence of serum, we reasoned that the kinin agonists should either originate from kininogens molecules bound to the surface of macrophages [46] or alternatively from kininogen molecules eventually sequestered from serum by promastigotes. To test the latter possibility, we washed stationary phase* L. major* promastigotes extensively as described for infection assays and then stained the parasites with two different domain-specific mAbs: (i) MBK_3_, a monoclonal antibody that recognizes the BK epitope (domain D_4_) of kininogens (HK/LK), and (ii) HKH_4_, a mAb that recognizes domain D_1_ of HK/LK [36]. FACS analysis showed that almost 70% of Δ*isp*2/3 promastigotes are positive stained for HKH4, compared to less than 50% of the WT parasites (Figure 3(a)). Along similar lines, MBK_3_ antibody showed that the BK epitope of kininogens was present in a higher proportion of Δ*isp*2/3 promastigotes as compared to WT promastigotes (Figure 3(b), 82,4%) as compared to WT parasites (Figure 3(b), 73.1%). These results suggest that both ISP-expressing* L. major* promastigotes and Δ*isp*2/3 mutants are able to sequester kininogens (retaining the intact BK molecule) from FCS. According to our working hypothesis (Figure 5), the kininogen opsonins tethered on ISP-deficient parasites may be cleaved by pericellular serine proteases (S_1a_ family) of macrophages, whereas the surface-bound kininogens associated to ISP-expressing (WT)* L. major* should be protected from proteolytic cleavage.

### 3.4. Targeting Activation of the Contact Phase/KKS in Human Plasma with Recombinant ISP-2 or Synthetic Inhibitor of Neutrophil Elastase

**Figure 4:**
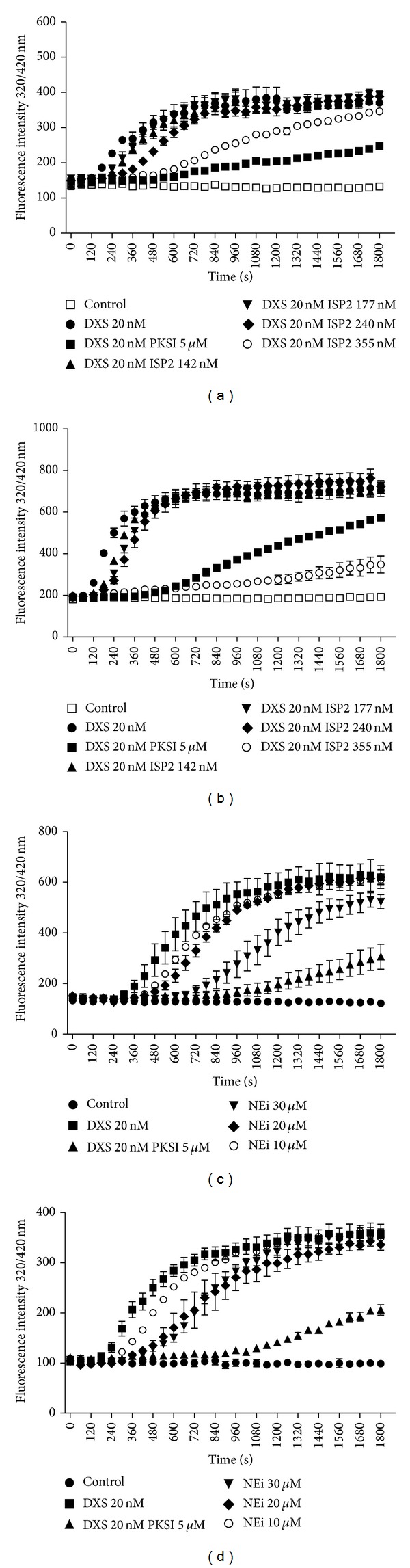
Effect of ISP2 on DXS-induced contact phase activation of human plasma. Citrated human platelet free plasma diluted 1 : 20 in buffer (described in methods) was supplemented with (i) 4 *μ*M Abz-MTEMARRPQ-EDDnp (a, c) or 4 *μ*M Abz-GFSPFRSVTVQ-EDDnp (b, d), intramolecular quenched fluorescent substrates whose sequences span the N-terminal or the C-terminal flanking sites (resp.) of BK in mHK (ii) dextran sulfate 500 kDa (DXS; 20 nM). The substrate was also tested in the absence of DXS (Control). Assays with the elastase inhibitor (NEi—MeOSuc-AAPV-CMK-10, 20, and 30 *μ*M), the synthetic PKa inhibitor (PKSI-527—5 *μ*M), and the inhibitor of serine peptidase 2 (ISP2—142, 177, 240, and 355 nM) were performed using two different schemes. (a, b) PKSI or the elastase inhibitor was added to the plasma together with DXS and the substrate. (c, d) PKSI or ISP2 was preincubated with plasma for 15 min, at 37°C, prior to the addition of DXS and the substrate. Hydrolysis was followed by measuring the fluorescence at *λ*_ex._ = 320 nm and *λ*_em._ = 420 nm (up to 1800 seconds). The plot shows the increase of fluorescence with time, reflecting substrate hydrolysis. The values in the figures represent the mean ± SE of duplicate determinations performed within 1 representative experiment of 2.

Considering that ISP-2 is hardly detected in the supernatants of* L. major* promastigotes (A. P. C. A. Lima, personal communication), we reasoned that we reasoned that this ecotin-like inhibitor may target S_1A_ serine proteases within the secluded spaces formed by the juxtaposition of host cell/parasite plasma membranes. Given the technical obstacles to monitor KKS activation and kinin release in this intercellular compartment, we first asked whether soluble (recombinant) ISP-2 or MeOSuc-AAPV-CMK (NE inhibitor) could inhibit the activation of the human contact system by DXS. This was addressed using a novel enzymatic assay that we recently used to detect the* P. duboscq* sandfly protein inhibitor of the contact system [25]. Briefly, the addition of DXS (500 kDa) to human plasma induces the reciprocal activation of FXII/PK, leading to the accumulation of PKa, the major kinin-releasing (S_1A_ family) in the plasma. Using as-read-outs synthetic substrates spanning the N-terminal or C-terminal flanking sequences of BK in the kininogen molecule, the kinetic measurements shown in our positive controls (Figure 4) reflect DXS-induced hydrolysis of the kininogen-like substrate by PKa [25]. Internal controls run in the presence of the synthetic PKa inhibitor (PKSI-257) show, as expected, pronounced inhibition of the contact phase enzyme by DXS. Assays performed with soluble ISP-2 revealed that the onset of hydrolysis was consistently delayed, in dose-dependent manner (range 142–355 nM; Figures 4(a) and 4(b)). A similar trend was observed when we added MeOSuc-AAPV-CMK (NE inhibitor) to the citrated plasma (range 10–30 *μ*M; Figures 4(c) and 4(d)). Collectively, these findings are consistent with the proposition that the activity of the contact phase enzyme complex (FXIIa/PKa) is at least partially inhibited by ISP-2 (soluble) or by the synthetic NE inhibitor.

## 4. Discussion

In the current study, we used genetically modified Δ*isp*2/3 mutants of* L. major* to determine whether these ecotin-like inhibitors regulate the proinflammatory activity of the kinin/B_2_R pathway* in vivo* and* in vitro*. In the first group of studies, we demonstrated that* L. major* promastigotes potently evoke plasma leakage and induce leukocyte accumulation in microvascular beds through mast cell-dependent activation of the kinin/B_2_R pathway, irrespective of the presence or absence of ISP-2. Extending this analysis to* in vitro *infection models, we showed that antagonists of B_2_R (HOE-140) or B_1_R (DAL^8^-BK) efficiently reversed the upregulated phagocytic uptake of* L. major *Δ*isp*2/3 by TG-macrophages without interfering with the internalization of ISP-expressing (WT) promastigotes. As discussed further below, these findings suggested that, upon attachment to the macrophage surface, ISP-expressing promastigotes might suppress the activation of B_2_R/B_1_R-dependent proinflammatory responses by inhibiting the kinin-releasing activity of serine proteases (S_1A_ family).

While studying the functional interplay between NE and ISP-2 during macrophage infection by* Leishmania*, Faria et al. [31, 32] demonstrated that two prominent phenotypes of Δ*isp*2/3 mutants (upregulated phagocytosis and induction of ROS via the elastase/TLR_4_/PKR pathway) were completely reversed in cultures supplemented with three different inhibitors of serine peptidases: aprotinin [27], a non-specific inhibitor of Arg-hydrolyzers, MeOSuc-AAPV-CMK (NE inhibitor), and the* L. major* ISP-2 (soluble/recombinant). Given the precedent that NE and mast cell tryptase (acting cooperatively) liberate bioactive kinin from oxidized kininogens [7], our findings that HOE-140 and DAL^8^-BK also reversed the phenotype of Δ*isp*2/3 mutants suggest that ISP-expressing* L. major* might inhibit the pericellular processing of surface-bound HK through the targeting of NE. Alternatively, our finding that soluble ISP-2 (or the NE inhibitor MeOSuc-AAPV-CMK) partially inhibit DXS-induced activation of the contact system in human citrated plasma (i.e., PKa-mediated hydrolysis of the flanking sites of BK in kininogen-like substrates) suggests that ISP-expressing promastigotes might rely on their surface-associated ISP-2 to target the contact phase enzymatic complex (FXIIa/PKa/HK) assembled on macrophage surfaces [46]. Admittedly, genetic studies will be required to dissect whether ISP-2 subverts innate immunity by protecting surface kininogens from the kinin-releasing activity of NE and/or by targeting surface assembled contact phase peptidases (FXII/PK). Although we have not systematically analyzed the impact of pharmacological blockade of B_2_R/B_1_R on the intracellular parasitism of TG-macrophages, preliminary results suggest that HOE-140 (tested at 100 nM) upregulates the outgrowth/survival of Δ*isp*2/3 mutants in TG-macrophages. If confirmed by genetic studies, our results may imply that* L. major* promastigotes might limit ROS formation via the NE/TLR_4_/PKR/TNF-*α*/IFN-*β* pathway originally described by Faria et al. [32] through ISP-2-dependent targeting of kinin-releasing peptidases assembled at the surface of macrophages.

A key event in many inflammatory processes is the adhesive interaction of circulating neutrophils and activated endothelial cells in postcapillary venules, a process that is often coupled to increased microvascular permeability, which in turn leads to the progressive accumulation of protein-rich edema fluid in interstitial tissues. Although conceding that the dynamics of the inflammatory responses that sand-fly-transmitted* Leishmania* induces in the injured dermis is far more complex than what is described in our intravital microscopy studies, the analysis of microvascular leakage and leukocyte accumulation in HCP topically sensitized with* L. major* promastigotes (WT or ISP2/3-deficient parasites) revealed that these parasites are far more potent inducers of plasma leakage and leukocyte accumulation than* L. donovani* [15],* L. chagasi* promastigotes (Figure 1(c)), or* T. cruzi* trypomastigotes [35]. Although the mechanisms underlying the discrepant phenotypes of* L. major* and* L. chagasi* or* T. cruzi* remain unknown, we were intrigued to find out that a 3X-fold higher dose of* L. chagasi* promastigotes did not evoke such a strong microvascular response, not even after treating the HCP with captopril, an inhibitor of kinin degradation by angiotensin-converting enzyme (Figure 1(c), black curve). In contrast,* T. cruzi* and* L. chagasi* are potentially lethal pathogens that disseminate systemically and preferentially target tissue in organs irrigated by fenestrated capillaries. Under these circumstances, plasma-borne substrates, such as kininogens, diffuse freely into the visceral tissues invaded by these visceralizing species of pathogenic trypanosomatids, both of which were empowered with kinin-releasing cysteine proteases [15, 47–49]. It is noteworthy that B_2_R-deficient mice acutely infected by* T. cruzi* [14] or* L. chagasi* [16] display heightened disease susceptibility, implying that the activation of the kinin/B_2_R pathway may preferentially shift the host/parasite balance towards protective immunity, at least during the acute phase.

Since proboscis inevitably provokes some extent of bleeding, we may predict plasma proteins and anti-inflammatory substances derived from the insect saliva are rapidly mixed with metacyclic parasites. Our studies in HCP topically sensitized with* L. major* promastigotes (which prevents bleeding and KKS activation due to pathogen inoculation through needles) suggest that these parasites potently evoke plasma leakage and leukocyte accumulation in microvascular beds via the kinin/B_2_R pathway. For reasons that are unclear, we found that Δ*isp*2/3 promastigotes evoked a somewhat milder inflammatory response (20%). Incidentally, Eschenlauer et al. [27] have reported that mice subcutaneously infected with* L. major* promastigotes transiently displayed higher tissue burden of ISP2/ISP-3-deficient parasites as compared to WT parasites. Lasting 3 days, the parasite burden subsequently equalized, implying that the selective advantage conferred to ISP-deficient promastigotes has waned as the infection progressed.

Based on pharmacological approaches, we showed evidences that* L. major* activates the KKS via mechanisms that involve transcellular cross-talk between neutrophils (intravascularly) and mast cells, a subset of innate sentinel cells that are mostly localized in perivascular tissues. Beyond the vasoactive role of histamine, a potent inducer of vascular permeability, mast cells also release heparin and polyphosphates, both of which were recently characterized as endogenous activators of the contact system [50, 51]. Given the interdependent nature of inflammatory circuits, it is likely that mast cells and the KKS/complement cascades are reciprocally activated and fueled in the HCP sensitized with* L. major* promastigotes. Although we have not studied the impact of the influx of complement into peripheral sites of* L. major* infection, it is well-documented that* Leishmania* lipophosphoglycan is opsonized by C3bi [52]. In the absence of other potent inflammatory cues, the engagement of macrophage CR3 by ISP-expressing* L. major* (WT) promastigotes may drive phagocytosis without necessarily stimulating the production of reactive oxygen intermediates, thereby creating a hospitable environment for the intracellular growth of* L. major* [31, 32]. Although studies in CD11b-deficient BALB/c mice have recently confirmed that activation of the C3bi/CR3 pathway increases host susceptibility to* L. major* infection [53], it will be interesting to know whether CR3-dependent suppression of IL-12 responses might depend on parasite-evoked extravasation of complement components to the extravascular compartment, as proposed here for plasma-borne kininogens.

**Figure 5:**
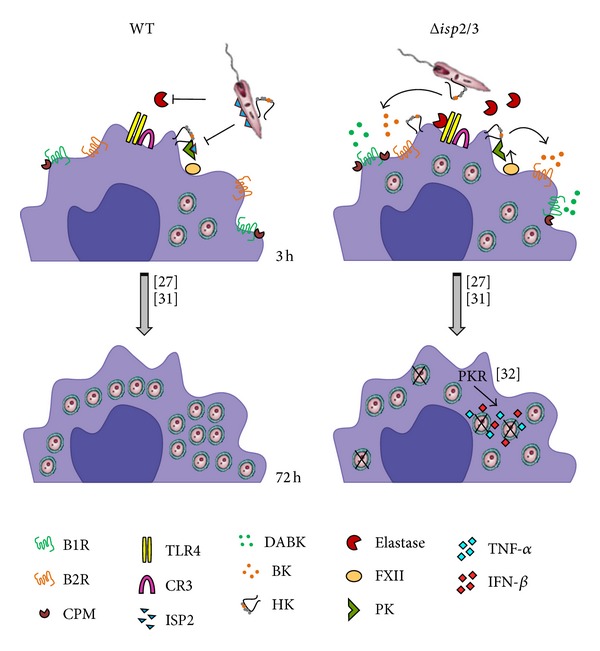
Scheme shows how ISP-2 limits the kinin-releasing activity of surface S_1_A-proteases of macrophages. As an extension of recently published studies [27, 31, 32], here we propose that the proinflammatory phenotype of the Δ*isp*2/3 mutant (right side of scheme) is due to increased pericellular release of kinins mediated by NE and/or contact phase serine proteases (FXIIa/PKa). In the absence of ISP-2, the “eat me signal” of kininogen tethered on* L. major* mutants might be inactivated by S_1_A-family proteases. In addition, the released kinin peptides fuel phagocytosis and microbicidal function of macrophages via activation of B_2_R and B_1_R, a subtype of GPCR upregulated in inflamed tissues.

Studies in the mouse ear model of sandfly-transmitted infection showed that* L. major* metacyclic promastigotes deposited in the dermis are engulfed by the infiltrating neutrophils within approximately 3 h [19–21]. Based on the results described in HCP topically sensitized with* L. major* promastigotes, it is conceivable that the proteolytic release of vasoactive kinins may further stimulate the transendothelial migration of neutrophils at very early stages of the infection. Furthermore, given evidence that parasitized neutrophils expose the apoptotic markers required for efferocytosis, it will be interesting to know whether plasma leakage may contribute to DC efferocytosis and to the ensuing suppression of Th1-inductive functions of DCs in the draining lymph nodes [19–21]. Beyond the impact on adaptive immunity, it is well established that efferocytosis of apoptotic neutrophils has profound effect on parasite survival in infected macrophages [22]. In this context, our finding that kininogen epitopes (N-terminal D1 and internal/BK/D4 domain) are tethered at the surface of* Leishmania* promastigotes is an intriguing finding because it raises the possibility that ISP-expressing promastigotes might “protect” the integrity of surface bound kinin precursors from premature proteolytic degradation by host proteolytic enzymes. This hypothesis is worth exploring in light of recent studies showing that HK (an abundant protein in the bloodstream; 660 nM) binds to PS exposed on apoptotic neutrophils before stimulating uPAR-dependent efferocytosis by macrophages via the p130Cas-CrkII-Dock-180-Rac1 pathway [6]. In other words, ISPs may protect the integrity of HK opsonins “eat me signals” while at the same time preventing the liberation of proinflammatory kinins (see scheme, Figure 5) within sites of parasite attachment to phagocytes. Although we have not explored the potential significance of* L. major* opsonization by kininogens, it will be interesting to know whetherparasite subsets bearing the uPAR ligand HK/HKa “eat me signal” might render macrophage permissive to intracellular survival, perhaps reminiscent of the apoptotic mimicry paradigm originally described by Barcinski and coworkers [54].

## 5. Conclusions

Extending the breadth of our previous investigations about the role of the kallikrein-kinin system in the immunopathogenesis of experimental Chagas disease and visceral leishmaniasis, the studies reported in this paper suggest that ecotin-like inhibitors expressed by* L. major *promastigotes fine-tune phagocytosis and may limit amastigote survival by inhibiting the pericellular activity of kinin-releasing serine proteases (S_1a_ family) of macrophages.
